# In Vitro Evaluation of Drug–Drug Interaction Potential of Epetraborole, a Novel Bacterial Leucyl-tRNA Synthetase Inhibitor

**DOI:** 10.3390/ph17010120

**Published:** 2024-01-17

**Authors:** Afshin Shafiee, Sanjay Chanda

**Affiliations:** AN2 Therapeutics Inc., 1800 El Camino Real, Suite D, Menlo Park, CA 94027, USA

**Keywords:** epetraborole, drug–drug interaction, bacterial leucyl-tRNA synthetase, *Mycobacterium avium* complex, melioidosis, in vitro studies

## Abstract

Epetraborole (EBO) is a boron-containing inhibitor of bacterial leucyl-tRNA synthetase, with potent activity against nontuberculous mycobacteria (NTM) and Gram-negative bacteria, including *Burkholderia pseudomallei*. EBO is being developed for the treatment of NTM lung disease and melioidosis, administered in combination with other therapeutic agents in both diseases. Therefore, EBO and its major circulating metabolite M3 were evaluated in comprehensive drug–drug interaction (DDI) in vitro studies. The CYP inhibitory and substrate potential of EBO and M3 were assessed using hepatic microsomes. Stably transfected cells that expressed individual efflux or uptake transporters were used to determine whether EBO or M3 were substrates or inhibitors for these receptors. Stability studies indicated that EBO is a poor substrate for major CYP enzymes. Neither EBO nor M3 was a potent reversible or time-dependent inhibitor of major CYP enzymes. EBO was not an inducer of CYP1A2 mRNA, while it was a weak inducer of CYP2B6 and CYP3A4. EBO was a substrate only for OCT2. At clinically relevant concentrations, neither EBO nor M3 inhibited major human efflux or uptake transporters. Based on these data, at clinically relevant concentrations of EBO and M3, there is a low risk of victim or perpetrator DDI.

## 1. Introduction

**Figure 1:**
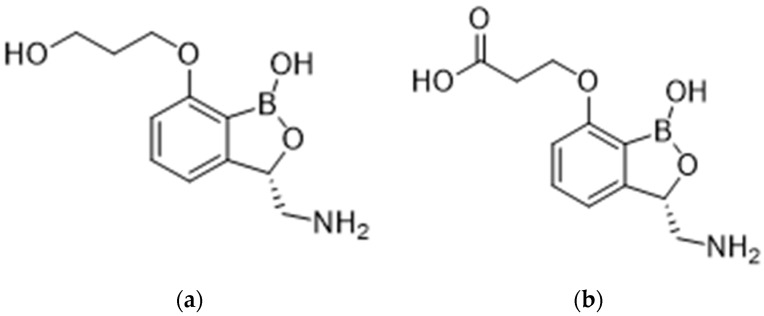
Chemical structures of (**a**) Epetraborole; (**b**) M3, the major metabolite of Epetraborole.

Epetraborole (EBO) is a unique aminomethyl benzoxaborole antibiotic that has broad-spectrum activity against the most common causative pathogens of nontuberculous mycobacteria (NTM) lung disease as well as Gram-negative bacteria, including the causative agent for melioidosis, *Burkholderia pseudomallei*. EBO has a novel mechanism of action that involves the inhibition of bacterial leucyl-tRNA synthetase, an aminoacyl-tRNA synthetase, which catalyzes an essential step in bacterial protein synthesis. EBO is highly distributed into tissues, and it is metabolized by oxidation of the propanol side chain to the corresponding carboxylic acid to form the inactive metabolite M3, potentially mediated by alcohol dehydrogenase (ADH) (Figure 1) [1].

Oral EBO is being developed for the treatment of serious infections for which there is a high unmet need for new antimicrobial therapy in combination regimens [2,3,4,5]. Currently, an oral formulation of EBO is in a Phase 2/3 clinical trial to investigate its safety and efficacy in treatment-refractory *Mycobacterium avium* complex (MAC) lung disease, the most common cause of NTM pulmonary lung disease [6]. The current standard of care (SoC) for MAC consists of chronic treatment with a macrolide (clarithromycin/azithromycin) in combination with rifampin and ethambutol lasting 18–24 months; other antibacterial therapies, including inhaled or intravenous amikacin, have been added to the treatment regimen with various degrees of success [7]. Approximately 35% of patients do not respond to the initial recommended treatment regimen [8], and no standard treatment regimen exists for the treatment-refractory disease [7,9]. Oral administration of EBO (500 QD) has shown a generally acceptable safety profile in healthy individuals and may be a viable addition to MAC treatment regimens [10].

The parenteral formulation of EBO is being evaluated for the treatment of acute melioidosis, a serious neglected tropical disease in Southeast Asia. Current SoC includes the use of broad-spectrum β-lactam antibiotics, ceftazidime or meropenem, administered intravenously for at least 10 days, followed by oral trimethoprim-sulfamethoxazole for 3 to 6 months [11,12,13,14]. The mortality rate for melioidosis remains high (>40%) with the current SoC, and the addition of another antibiotic with potent activity against *Burkholderia pseudomallei*—like epetraborole—has the potential to reduce the death rate in these patients [15].

Because EBO is being developed for NTM lung disease and melioidosis where it will be administered in combination with other therapeutic agents, the present study assessed the in vitro drug–drug (DDI) interaction potential of EBO and its metabolite, M3, in accordance with the US Food and Drug Administration (FDA) guidelines [16]. These in vitro studies may not fully represent the complexities of DDI in vivo, but the in vitro as well as human pharmacokinetics data determine if a DDI clinical study is warranted.

## 2. Results

### 2.1. EBO Permeability in Caco-2 Cell Monolayer

The integrity of the Caco-2 cell monolayers was confirmed by measuring TEER before and after incubation. The apparent permeabilities obtained with mannitol (a paracellular marker) and caffeine (a transcellular marker) and the efflux ratio obtained with digoxin (a P-gp substrate) in the absence and presence of a known P-gp inhibitor (cyclosporine A at 10 μM or verapamil at 100 μM) demonstrated that the Caco-2 cell monolayers functioned normally. The apparent permeabilities of mannitol and caffeine were 0.19 to 0.69 × 10^−6^ cm/s and 31.0 to 39.4 × 10^−6^ cm/s, respectively, in the apical to basolateral (A to B) or basolateral to apical (B to A) directions (efflux ratio). The efflux ratio of digoxin ranged from 7.07 to 9.07 in the absence of a P-gp inhibitor and was reduced to a range of 0.69 to 2.11 in the presence of cyclosporine A (10 μM) or verapamil (100 μM).

The apparent permeability of EBO (1, 10, and 100 µM) through Caco-2 monolayers was determined in both the A to B and B to A directions, in the absence and presence of a P-gp inhibitor [cyclosporine A (10 μM) or verapamil (100 μM)]. In the absence of a P-gp inhibitor, the apparent permeabilities of EBO were ≤0.31 × 10^−6^ cm/s in the A to B direction and ≤0.35 × 10^−6^ cm/s in the B to A direction with efflux ratio <2. In the presence of a P-gp inhibitor, the efflux ratio was not changed significantly; therefore, EBO is not a P-gp substrate. In addition, EBO was not a significant inhibitor of the P-gp-mediated transport of digoxin (Table 1). The efflux ratio of digoxin (7.07) was unchanged by the presence of EBO 100 µM.

### 2.2. Transporter Substrate

At 5 µM, in MDR1-MDCK, BCRP-MDCK, and MDCK cells, the concentration of EBO or M3 in the receiver compartment was below the lower limit of quantification in both the A to B and B to A directions; therefore, the apparent permeability coefficient (Papp) and efflux ratio of EBO could not be calculated in either cell line, and the net flux ratio of EBO was not applicable. Efflux ratios for control compounds digoxin and cladribine were 244 and 288 for the respective test systems, indicating the functional expression of P-gp and BCRP.

EBO and M3 were not substrates for OAT1, OAT3, MATE 1, and MATE2K, OATP1B1, and OATP1B3 with influx ratios of <2. EBO (5 µM) had an influx ratio of 3.44 and could potentially be a substrate for OCT2, while M3 was not a substrate (Table 2).

### 2.3. Transporter Inhibition

EBO was soluble up to 17,000 µM and 100 µM in transport buffer at pH 7.4 and pH 8.5, respectively. M3 was soluble up to 250 µM at pH 7.4 and pH 8.5. Neither EBO nor M3 induced any cytotoxicity at the concentrations tested.

In the absence of a P-gp inhibitor, the efflux ratio of digoxin was 131; the addition of valspodar decreased it to 1.00, corresponding to complete inhibition. These results indicate the normal function of P-gp in MDR1-MDCK cells. In the presence of EBO, the efflux ratio of digoxin ranged from 46.6 to 269. Only at the highest concentration tested (17,000 µM), EBO showed >50% inhibition of digoxin efflux transport with an IC_50_ value of 5651 µM (Table 2). EBO and M3 weakly inhibited BCRP-mediated efflux transport at 300 and 1000 μM (13 and 27%, respectively, for EBO) and at 500 μM (44% for M3) and inhibited the human OATP1B1 (25%) and OAT1B3 (21%) at 500 μM. However, the data were insufficient for the calculation of IC_50_ values. EBO and M3 did not inhibit the human renal uptake of OAT1 or OAT3 transporters at concentrations up to 1000 μM. EBO inhibited the transport of metformin via human OCT1 (59%) and OCT2 (20%) at the maximum concentration tested, 1000 μM. EBO IC_50_ values were >100 µM for MATE1 and MATE2K (Table 2).

### 2.4. CYP Induction

EBO and M3 were soluble in hepatocyte incubation medium at 20 mM and 250 µM, respectively, and did not induce cytotoxicity (>20%) at 100 µM or 250 µM for EBO and M3, respectively.

At 100 µM, EBO showed induction potential for CYP2B6 (2.18-fold) and CYP3A4 (~3-fold) in mRNA expression levels in 1 out of 3 and 2 out of 3 donors, respectively. However, at the tested concentration range, the weak induction response did not allow the determination of an EC_50_ value for CYP 2B6 and 3A4, at the highest EBO concentration evaluated. At 100 and 250 µM, M3 showed induction potential for CYP3A4 mRNA expression levels in 1 out of 3 donors (2.19- and 2.13-fold, respectively). M3 induced CYP3A4 mRNA expression with an E_max_ value of 2.16-fold and an EC_50_ value of 32.44 µM in the same donor (Table 3).

### 2.5. CYP Inhibition

EBO weakly inhibited CYP1A2 activity with a decrease of approximately 30% at the highest concentration tested (100 µM) with no effect on the other CYP enzymes studied (Table 4). Preincubation of EBO with hepatic microsomes without NADPH produced inhibition congruent with co-incubation data, with a mild decrease in CYP1A2 activities. The percentages of activity remaining for CYP1A2 activities were 72.4% and 81.7% upon incubation with EBO in the absence and presence of NADPH, respectively. No time-dependent inhibition of CYP3A4 or CYP2D6 with EBO (30 and 50 µM) was noted. No direct or time-dependent inhibitory activity of M3 on CYP1A2, CYP2D6, CYP3A4, CYP2C8, or CYP2C9 was observed.

In vitro metabolism studies of M3 were not conducted because results from a human study of radiolabeled EBO indicate that M3 is not subject to further metabolism once formed, and is excreted predominantly in the urine, with a minor amount in feces [17].

## 3. Discussion

Based on the chemical structure and novel mechanism of action and its planned use as part of combination regimens in patients, EBO’s potential for DDI needs to be investigated fully. These data are intended to support the use of oral and intravenous EBO in the treatment of NTM lung disease and melioidosis, respectively, in combination with SoC therapy. The addition of a novel agent with reduced DDI potential to existing SoC antibiotics could significantly reduce the risk of new DDI-emergent side effects.

EBO appears to have a low DDI potential due to a lack of observed metabolism by the CYP enzymes, the major metabolizing enzyme system for a wide range of therapeutic drugs. Furthermore, in this study, we demonstrated that neither EBO nor M3 caused potent induction of CYP1A2, 2B6, and 3A4 in human hepatocytes nor significantly inhibited their activity. NTM lung disease patients may be prescribed combination regimens for up to 24 months that include agents such as clarithromycin/azithromycin, rifampin, and ethambutol, which have been shown to cause DDI mediated by inhibition or induction of CYP enzymes. Rifampin is an inducer of CYP3A4 and CYP2Cs as well as efflux protein P-gp [18]. Clarithromycin has been associated with significant DDI by inhibiting CYP3A4 and uptake transporter OATP1B1 [19,20]. Ethambutol is also a potent inhibitor of CYP1A2 and CYP2E1 and a moderate inhibitor of CYP2C19 and CYP2D6 and it is an inhibitor of OCT1 and OCT2, which would alter absorption, distribution, and excretion of co-administered cationic drugs [21,22]. Meropenem is a low-affinity substrate for OAT1 and OAT3 and a weak inhibitor of uptake and efflux transporters [23].

EBO exhibits low permeability and is not a substrate of the human P-gp transporter protein in vitro, which suggests minimal to no effect on the absorption and disposition of EBO in patients. Neither EBO nor M3 were substrates for the major human uptake transporters, with the exception of OCT2, a transporter that is involved in active renal secretion [24]. However, renal clearance of EBO in human subjects is approximately 110 mL/min (6.62 L/h), a value that is comparable to the glomerular filtration rate and indicates that active renal secretion is not a major contributor to clearance [25]. EBO was a weak inhibitor of efflux transporters P-gp (IC_50_ = 5651 µM) BCRP and uptake transporters OATP1B1, OAT1B3, OCT1, and OCT2 at concentrations markedly higher than systemic exposures at clinically relevant doses with potentially no effect on EBO’s disposition or efficacy.

In a Phase 1 dose-ranging study, Study EBO-101, following administration of EBO 500 mg PO q24h to healthy subjects—the clinical dosage being administered in an ongoing MAC lung disease trial [26]—steady-state systemic total C_max_ of EBO and metabolite M3 were 12 µM and 20 µM (unbound fraction), respectively [10]. The calculated maximum intestinal concentration at a dose of 500 mg (dose divided by 250 mL) of EBO is approximately 8500 µM. This implies a low risk of drug interactions due to P-gp since the [I_gut_]/IC_50_ = 8500 µM/5651 µM is ≤3, which is well below ≥10 K_i_ value required by the US FDA guidance [16] for a drug to be considered for a DDI clinical study. Based on the in vitro data from different transporter-mediated interactions, C_max_ of 2.85 μg/mL (approximately 12 μM) from a 500 mg/day PO human clinical dose for MAC lung disease, the potential for clinically significant interactions with EBO is predicted to be low.

## 4. Materials and Methods

EBO and M3 were synthesized by Regis Technologies (Morton Grove, IL, USA), Acme (Palo Alto, CA, USA), Esteve Química (Barcelona, Spain), Chemical Development GlaxoSmithKline (King of Prussia, PA, USA)] and ^14^C-EBO was synthesized by GE Healthcare (Cardiff, UK).

Caco-2 cells were purchased from the American Type Culture Collection (Manassas, VA, USA). The HEK 293-OATP1B1, -OATP1B3, -OAT3, -NTCP, and -ASBT stable cells and mock cells were purchased from Corning Life Science (Woburn, MA, USA). Characterized, pooled, human hepatic microsomes from 15 individuals (10 males and 5 females) were obtained from CellzDirect (Durham, NC, USA). Pooled human liver microsomes from 15 donors were obtained from XenoTech LLC, Lenexa, KS, USA. Cryopreserved plateable human hepatocytes were purchased from BioIVT (Baltimore, MD, USA).

Atenolol, digoxin, propranolol, atorvastatin, furosemide, 1-methyl-4-phenylpyridinium iodide (MPP+), para-aminohippurate (PAH), metformin, fluorescein methotrexate (FMTX), 4-(4-dimethylaminostyryl)-N-methylpyridinium (ASP), 5-carboxyfluorescein (5-CF), 6-carboxyfluorescein (6-CF), clofarabine, Ko143, estrone-3-sulfate (E3S), geneticin (G418), puromycin, repaglinide, valspodar, omeprazole, phenobarbital, rifampin, flumazenil and D-glucose were obtained from MilliporeSigma (St. Louis, MO, USA) and Toronto Research Chemicals Inc. (North York, ON, Canada). Cladribine and deuterated internal standards (atorvastatin-d5, MPP+-d3, furosemide-d5, and metformin-d6) were purchased from Toronto Research Chemicals (Toronto, ON, Canada). Para-aminohippurate (PAH-d4) was purchased from C/D/N isotopes (Quebec, QC, Canada). Lucifer yellow (LY), HEPES, Hank’s balanced saline solution (HBSS), Dulbecco’s modified Eagle’s medium (DMEM), and Dulbecco’s phosphate-buffered saline (DPBS) were obtained from Life Technologies (Carlsbad, CA, USA). Fetal bovine serum (FBS) was purchased from ThermoFisher Scientific (Waltham, MA, USA). Dimethyl sulfoxide (DMSO), acetonitrile, isopropanol, methanol, and ammonium hydroxide were purchased from EMD Chemicals (Darmstadt, Germany). Formic acid was purchased from Avantor Performance Materials (Center Valley, PA, USA). Penicillin (100 IU/mL) and streptomycin (100 µg/mL) mixture (PEST), nonsessential amino acids (NEAA), and trypsin were obtained from CellGro (Herndon, VA, USA). Transwell^®^ 12-well plates were purchased from Corning Life Sciences (Corning, NY, USA). The BCA (bicinchoninic acid) Protein Assay kit was purchased from Thermo Fisher Scientific Inc. (Rockford, IL, USA). Poly-D-lysine 24-well multiwell plates were obtained from BD Gentest (Woburn, MA, USA). Radioimmunoprecipitation (RIPA) buffer was purchased from Santa Cruz Biotechnology (Santa Cruz, CA, USA). cDNA Reverse Transcription kits, TaqMan primers and probes, and TaqMan PCR Master Mix kits were obtained from Applied Biosystems Life Technologies (Grand Island, NY, USA). CellTiter-Glo Assay kit and Total RNA Isolation Systems were purchased from Promega (Madison, WI, USA).

### 4.1. In Vitro Effect of EBO and M3 on Efflux and Uptake Transporters

^14^C-EBO permeability in Caco-2 Cells (MDR1/Protein Glycoprotein or P-gp) was determined using methodologies described previously [27,28,29,30,31]. The apparent permeability of ^14^C-EBO was determined in both the apical to basolateral and the basolateral to apical directions, in triplicate, at final concentrations of 1, 10, and 100 μM. Cell monolayers were incubated with ^14^C-EBO at 37 °C for 1, 2, 3, and 4 h. The transepithelial electrical resistance (TEER) values of the monolayers prior to and following each experiment were measured to confirm the integrity of the Caco-2 cell monolayers. The apparent permeability of ^14^C-EBO (at a final concentration of 10 μM) was determined in both the apical to basolateral and basolateral to apical directions under the same conditions as previously described in the presence of P-gp inhibitors cyclosporine A (10 μM) and verapamil (100 μM) in the donor compartment.

The effect of EBO (at final concentrations of 0, 0.2, 1, 5, 25, and 100 µM), cyclosporine A (10 μM), and verapamil (100 µM) on the P-gp mediated transport of ^3^H-digoxin (1 μM) in HBSS pH 7.4 was determined after incubation for 1 h in both the apical to basolateral and the basolateral to apical directions, in triplicate. The concentrations of each radiolabeled compound in the resulting samples were determined using liquid scintillation counting (LSC).

### 4.2. In Vitro Evaluation of the Substrate Potential of EBO and M3 for MDR1 (P-Glycoprotein; P-gp) or BCRP (Breast Cancer Protein Resistant Protein)

In vitro evaluation of the substrate potential of EBO (5 µM) and M3 (5 µM) for P-gp was conducted in single transporter-transfected and non-transfected Madin–Darby canine kidney (MDCK) cells monolayers according to Absorption Systems (Exton, PA, USA) standard operating procedures (SOP). The integrity of each cell monolayer was evaluated by lucifer yellow presence. A bidirectional permeability assay was conducted for each compound and control compounds digoxin and cladribine. For the test article group, a dosing solution (0.55 mL for AP-to-BL, 1.55 mL for BL-to-AP) was added to the donor compartment, and HBSS (1.5 mL for AP-to-BL, 0.5 mL for BL-to-AP) was added to the receiver compartment. Receiver samples (200 µL) and donor samples (50 µL) were taken at preselected time points. The concentrations of each test article, digoxin, and cladribine in the receiver and donor samples were determined by high-performance liquid chromatography–mass spectrometric (LC-MS/MS) methods.

### 4.3. In Vitro Evaluation of the Substrate Potential of EBO and M3 for, OATP1B1, OATP1B3, OAT1, OAT3, OCT2, MATE1, or MATE2K-FDA 2020

In vitro evaluation of the substrate potential of EBO (0.5 and 5 µM) and M3 (0.5 and 5 µM) for OATP1B1, OATP1B3, OAT1, OAT3, OCT2, MATE1, or MATE2K were evaluated in single transporter-transfected and non-transfected MDCK cells according to Absorption Systems (Exton, PA, USA) SOP. The uptake of a probe substrate of each transporter was conducted in parallel with the test article in separate wells of the same batch of vector control and transporter-transfected cells. Cells were incubated at 37 °C with 5% CO_2_ with 500 µL of dosing solution for different periods of time. At the end of the incubation period, the dosing solution was gently aspirated; the cells were rinsed twice with ice-cold HBSS (pH 7.4) buffer and lysed in acetonitrile:water (3:1, *v*/*v*) containing internal standard. The lysates (300 µL) were collected for determination of test article and probe substrate concentrations by LC-MS/MS methods.

### 4.4. In Vitro Inhibition Potential (IC_50_) Assessment of EBO and M3 as an Inhibitor of Efflux Transporters P-gp and BCRP

In vitro inhibition potential (IC_50_) assessment of EBO (50–17,000 µM) as an inhibitor of P-gp was carried out in MDR1-MDCK, BCRP-MDCK, and MDCK cells according to Absorption Systems (Exton, PA, USA) SOP. Bidirectional transport of digoxin (10 µM) was measured in the absence and presence of the test article or valspodar.

A preincubation with EBO or known inhibitor was performed on both sides to preload the cells. After 30 min, the preincubation solution was aspirated. Aliquots of fresh dosing solution with or without EBO or known inhibitor (0.55 mL for AP-to-BL, 1.55 mL for BL-to-AP) were added to the donor compartment, and HBSSg_7.4 with or without EBO or known inhibitor (1.5 mL for AP-to-BL, 0.5 mL for BL-to-AP) were added to the receiver compartment. Receiver samples (200 µL) and donor samples (50 µL) were taken at preselected time points.

Cell monolayer integrity was examined using lucifer yellow (excitation 450 nm and emission 538 nm). The concentrations of the probe substrate in the donor and receiver samples, as well as EBO in dosing solutions, were determined by LC-MS/MS.

### 4.5. In Vitro Inhibition Potential (IC_50_) Assessment of EBO and M3 as Inhibitors of Uptake Transporters

In vitro inhibition potential (IC_50_) assessment of EBO (0.27–1000 µM) or M3 (1.03–250 µM) as inhibitors of uptake transporters (OATP1B1, OATP1B3, OAT1, OAT3, OCT1, and OCT2) was carried out in either human embryonic kidney 293 (HEK 293) or MDCK cell lines transfected with each of the uptake transporters in 4 separate studies according to Absorption Systems (Exton, PA, USA), Optivia Biotechnology (Menlo Park, CA, USA), GlaxoSmithKline (King of Prussia, PA, USA or Ware, Hertfordshire, UK) SOP.

Cell monolayers were preincubated (37 °C) for 15 to 30 min in a transport medium containing the target concentration of EBO or a specific inhibitor without substrate. Following the removal of preincubation solutions, appropriate working solutions containing EBO at the target concentration plus the probe substrate were added to the wells in triplicate. Separate sets of triplicate wells were designated for probe substrate only and probe substrate plus the specific inhibitors, to determine the maximal uptake rate and maximal inhibition of uptake, respectively. After incubation at 37 °C for each transporter, the working solution was removed from each well and the experiment was stopped by washing three times with cold (4 °C) DPBS. Depending on the substrate label, cells were either lysed for the analysis of total radioactivity (liquid scintillation counter) or LC-MS/MS methods. For measuring relative fluorescence (FLUOstar Galaxy, Cary, NC, USA), cells were not lysed.

### 4.6. Potential CYP Induction by EBO and M3

The induction potential of EBO and M3 on CYP1A2, CYP2B6, and 3A4 was evaluated by incubating the test articles with plated human hepatocytes from 3 individual donors (GKJ, ZEY, WKF) in triplicate according to WuXi AppTec (Cranbury, NJ, USA) SOP. Hepatocytes were plated overnight and then they were treated for 2 days with EBO (0.3–100 µM) or M3 (1–250 µM), known inducers, or a known non-inducer. Omeprazole (50 µM), phenobarbital (750 µM), and rifampin (25 µM) were used as positive controls for the induction of CYP 1A2, 2B6, and 3A4, respectively. Flumazenil (25 µM) was used as the negative control (non-inducer) for all CYP isoforms.

At the end of incubation, the RNA of human hepatocytes was isolated and the mRNA expression levels of CYP 1A2, 2B6, and 3A4 were evaluated by using real-time polymerase chain reaction (qPCR) after being reverse-transcribed (RT) to cDNA following manufacturer’s instruction (thermos Fisher Scientific (Waltham, MA, USA). On the last dosing day with EBO or M3, incubation samples were extracted in triplicate by the addition of ice-cold quench solutions at 0, 0.5, 1, 2, 4, 6, and 24 h after dosing and the extracted samples were analyzed using LC-MS/MS to determine the EBO or M3 concentration in each sample.

### 4.7. Inhibitory Potential of EBO on Human Hepatic Microsomal Cytochrome P450

The direct inhibitory potential of EBO on cytochrome P450 activities O-deethylase (CYP1A2), bupropion hydroxylase (CYP2B6), amodiaquine N-deethylase (CYP2C8), diclofenac 4′-hydroxylase (CYP2C9), S-mephenytoin 4′-hydroxylase (CYP2C19), bufuralol 1′-hydroxylase (CYP2D6), chlorzoxazone 6-hydroxylase (CYP2E1), testosterone 6β-hydroxylase (CYP3A), and midazolam 1′-hydroxylase (CYP3A) was assessed in vitro using human hepatic microsomes according to Covance Laboratory (Madison, WI, USA) SOP. A single substrate concentration was used, approximating the concentration of substrate that gives half the maximum reaction velocity (Km) for human hepatic microsomes for each cytochrome P450 activity. Assays were performed in the presence and absence of EBO (0.03–100 μM) to determine its inhibition potential on selected cytochrome P450 activities. When >50% inhibition was observed, the IC_50_ for EBO was determined. All sample and control incubations were performed in triplicate and were analyzed by LC-MS/MS.

Incubation mixtures contained microsomal protein and substrate in a 0.1 M potassium phosphate buffer (pH 7.4) containing 1 mM EDTA (assay buffer). Microsomal protein was incubated at 37 °C for at least 3 min in the presence of substrate and EBO prior to the addition of warmed (37 °C) NADPH to initiate the reaction. Incubations were terminated by the addition of 100 µL of an internal standard solution followed by vortex mixing. Protein was removed by centrifugation at 1500× *g* for 5 min at 4 °C, and supernatants were transferred to a separate plate and stored at 4 °C prior to LC-MS/MS analysis. The analyte for each activity assay was quantitated by comparison to a linear curve of an authentic standard prepared using human microsomal protein.

Metabolism-dependent inhibition was assessed with human hepatic microsomes incubated with EBO (0.01, 0.1, 1, and 100 µM) or solvent and NADPH for 15 min prior to the addition of a cytochrome P450 marker substrate. Incubations were performed at substrate concentrations approximating the average Km values. Control incubations were performed with EBO (0.01, 0.1, 1, and 100 µM) or solvent in the absence of NADPH during the 15 min preincubation prior to the addition of a cytochrome P450 marker substrate. For determination of control activities, NADPH was added after the addition of each substrate. Samples were then processed as previously described.

### 4.8. Direct and Time-Dependent Inhibitory Potential of M3 on Human Hepatic Microsomal Cytochrome P450

Inhibition assay with M3 (1–1000 µM) was performed according to GlaxoSmithKline (King of Prussia, PA, USA) SOP. Incubation mixture (250 µL) consisted of 145 µL of incubation mixture (50 mM phosphate buffer pH 7.4 and microsomes) and 5 µL of M3 solution in water. Two sets of duplicate incubations were prepared (cofactor preincubation [50 µL] and control preincubation [50 µL]) with each concentration of M3. All incubations contained a final microsomal protein concentration of 0.1 mg/mL. In this study, M3 and microsomes were preincubated with cofactor for 20 min prior to initiation of reaction by addition of probe substrate. The corresponding control incubations were designed to provide a preincubation without cofactor while incorporating the 20 min preincubation of M3 with microsomes to ensure consistent equilibration of M3 in both control and cofactor preincubations. The control preincubations were therefore performed for 20 min with M3, microsomes, and probe substrate, and the reaction was initiated by the addition of a cofactor solution.

Positive control incubations (replacing M3 with an appropriate concentration range of a time-dependent CYP inhibitor) and control incubations without inhibitor (containing 2% water or 2% methanol only) were also performed. Incubations without cofactor (at the highest concentration of M3) were performed to determine any cofactor-independent substrate metabolism formation. Probe substrate concentrations were determined by LC/MS/MS methods.

### 4.9. Time-Dependent Inhibitory Potential of EBO on Human Hepatic Microsomal CYP3A and CYP2D6

The effect of EBO (30 and 50 µM) on time-dependent inhibition of CYP3A4 (testosterone and midazolam substrate) and CYP2D6 (dexamethasone substrate) was determined using methodologies described previously [32]. The Genesis robot (Tecan, Reading, UK) running Gemini software (https://www.tecan.com) was used to perform a fully automated TDI assay. The assay ran a preincubation (30 min at 37 °C, 1 mg/mL HLM incubation containing 1 mM NADPH and EBO) and a secondary incubation (containing 50 µL aliquot from the preincubation, 1 mM NADPH, and substrates for CYP3A4 and CYP2D6). At the end of the secondary incubation (15 min at 37 °C), an aliquot (50 µL) was removed and added to 100 µL of methanol to quench the reaction. Quenched samples were then chilled at −20 °C for 2 h, centrifuged at 2000× *g* for 15 min, and the supernatants were transferred to microtiter plates for LC/MS-MS analysis.

## 5. Conclusions

A comprehensive in vitro evaluation of EBO and its major metabolite, M3 was conducted following the US FDA guidance for in vitro-based transporter- and CYP-mediated DDI. Based on these data, at clinically relevant concentrations of EBO and M3, there is a low risk of victim or perpetrator DDI allowing for the chronic co-administration of EBO with the SoC antibiotics with reduced chance of DDI-emergent side effects. EBO is currently in Phase 2/3 clinical trials to evaluate its safety and efficacy in patients with MAC lung disease and clinical trials to investigate EBO’s safety in melioidosis patients are planned.

## Figures and Tables

**Table 1:** Efflux ratios from Caco-2 cells incubated with ^14^C-EBO.

^14^C-EBO (µM)	Treatment (µM)	Incubation Time (Hour)	Efflux Ratio
1, 10, 100	Vehicle	1–4	<2
	Cyclosporine A (10)	1–4	<2
	Verapmil (100)	1–4	<2

**Table 2:** Transporter-mediated drug interaction potential of EBO in human cell lines.

Transporter	EBO as an Inhibitor, IC_50_ (µM)	EBO as a Substrate	M3 as an Inhibitor, IC_50_ (µM)	M3 as a Substrate
OATP1B1	25% inhibition at 500 µM	No	>250	No
OATP1B3	21% inhibition at 500 µM	No	>250	No
OCT1	59% inhibition at 1000 µM	ND	>250	ND
OCT2	20% inhibition at 1000 µM	Yes	>250	No
OAT1	>1000	No	>250	No
OAT3	>1000	No	>1000	No
P-gp	5651	No	ND	No
BCRP	27% inhibition at 1000 µM	No	>500	No
MATE1	>100	No	>250	No
MATE2K	>100	No	>250	No

BCRP = breast cancer resistance protein; IC_50_ = concentration associated with 50% inhibition; ND = not determined; MATE = multidrug and toxin extrusion; OAT = organic anion transporter; OATP = organic anion transporting polypeptide; OCT = organic cation transporter; P-gp = P-glycoprotein.

**Table 3:** Cytochrome P450 enzyme induction potential of EBO and M3.

CYP	Donor	Test Article	Apparent E_max_ (Fold)	Apparent EC_50_ (µM)	mRNA Induction Fold in Controls	
					NC	PC
1A2	GKJ	EBO (0.3–100 µM)	<2	NA	0.94	32.07
	ZEY		<2	NA	0.75	32.54
	WKF		<2	NA	0.71	49.02
2B6	GKJ		>2.18	>100	1.09	6.88
	ZEY		<2	NA	1.00	6.64
	WKF		<2	NA	0.84	7.45
3A4	GKJ		>2.93	>100	0.96	87.53
	ZEY		<2	NA	0.98	78.29
	WKF		>2.98	>100	0.89	126.49
1A2	GKJ	M3 (1–250 µM)	<2	NA	0.82	31.62
	ZEY		<2	NA	0.74	29.34
	WKF		<2	NA	0.94	56.73
2B6	GKJ		<2	NA	0.87	6.29
	ZEY		<2	NA	0.87	6.86
	WKF		<2	NA	0.91	9.17
3A4	GKJ		<2	NA	0.66	84.87
	ZEY		<2	NA	0.87	91.32
	WKF		2.16	32.44	1.06	167.94

NC: Negative control—flumazenil (25 µM) was used as the negative control treatment. PC: Positive control: omeprazole (50 µM) was a positive control inducer for CYP1A2. Phenobarbital (750 µM) was a positive control inducer for CYP2B6. Rifampin (25 µM) was a positive control for CYP3A4. <2—No induction at tested concentrations. GKJ, ZEK, WKF: Initials of the donors. NA—not applicable. No induction was observed at the highest concentration tested (highest concentrations selected based on solubility and cytotoxicity) and regression for E_max_ and EC_50_ was not performed. Data are means from triplicate measurements.

**Table 4:** Inhibition potential of EBO and M3 in cytochrome P450 enzymes.

Enzyme/Transporter	EBO as an Inhibitor IC_50_ (µM)	M3 as an Inhibitor IC_50_ (μM)	MDI IC_50_ Fold Change
CYP1A2	100 (30%)	>1000	No
CYP2B6	>100	-	No
CYP2C8	>100	>1000	No
CYP2C9	>100	>1000	No
CYP2C19	>100	-	No
CYP2D6	>100	>1000	No
CYP2E1	>100	-	No
CYP3A4 (Testosterone)	>100	>1000	No
CYP3A4/5 (Midazolam)	>100	>1000	No
CYP3A4/5 (Atorvastatin)	-	>1000	No

CYP = cytochrome P450; IC_50_ = concentration associated with 50% inhibition; MDI = metabolism-dependent inhibition.

## Data Availability

The data presented in this study are available on request from the corresponding author. The data are not publicly available due to privacy.
